# Epidemiology of sepsis in hospitalised neonates in Indonesia: high burden of multidrug-resistant infections reveals poor coverage provided by recommended antibiotic regimens

**DOI:** 10.1136/bmjgh-2024-016272

**Published:** 2025-04-09

**Authors:** Nina Dwi Putri, Benjamin FR Dickson, Riyadi Adrizain, Leny Kartina, Jannah Baker, Distiayu Sukarja, Fabiola Cathleen, Dominicus Husada, Martono T Utomo, Tetty Yuniati, Adhi K Suginali, Michelle Harrison, Michael Sharland, Phoebe CM Williams

**Affiliations:** 1 Universitas Indonesia, Depok, Indonesia; 2 School of Public Health, Faculty of Medicine, The University of Sydney, Sydney, New South Wales, Australia; 3 Sydney Institute of Infectious Diseases, Faculty of Medicine, The University of Sydney, Sydney, New South Wales, Australia; 4 Department of Paediatric Infectious Diseases, Dr Hasan Sadikin Central General Hospital Bandung, Bandung, West Java, Indonesia; 5 Department of Paediatric Infectious Diseases, Dr Soetomo Regional General Hospital, Surabaya, East Java, Indonesia; 6 Department of Child Health, Dr Soetomo Regional General Hospital, Surabaya, East Java, Indonesia; 7 Dr Soetomo Regional General Hospital, Surabaya, East Java, Indonesia; 8 Microbiology Department, Dr Hasan Sadikin Central General Hospital Bandung, Bandung, West Java, Indonesia; 9 St George’s University of London Institute for Infection and Immunity, London, London, UK; 10 Sydney Childrens Hospital, Department of Infectious Diseases, University of New South Wales, Sydney, New South Wales, Australia

**Keywords:** Blood disorders, Child health, Infections, diseases, disorders, injuries, Public Health, Global Health

## Abstract

**Background:**

Neonatal sepsis is a leading cause of child mortality, and this burden is rising secondary to increasing antimicrobial resistance worldwide. There are marked global disparities in the burden of antimicrobial resistance, with Southeast Asia identified as a region of particular concern. Indonesia is the world’s fourth most populous country, with 4.2 million babies born each year. Despite this, there remains limited published data on the burden of neonatal sepsis and antimicrobial resistance in the country.

**Methods:**

We conducted a 2-year analysis of the epidemiology of neonatal sepsis across three high-burden clinical settings in Indonesia, alongside an antibiotic point prevalence prescribing survey.

**Results:**

Of 5439 blood cultures analysed, gram-negative bacteria predominated as causative pathogens for neonatal sepsis in Indonesia, with *Klebsiella* spp and *Acinetobacter* spp most common (prevalence 35% and 19%, respectively). Overall, the pathogens causing neonatal sepsis exhibit very low susceptibility to WHO-recommended antibiotic regimens for the treatment of neonatal sepsis, with only 25% coverage provided by aminopenicillins plus gentamicin (95% credible interval (CI) 22% to 29%) and 20% by third-generation cephalosporins (95% CI 17% to 23%). Almost half of all antibiotics prescribed to treat neonatal sepsis across the clinical settings included in our study were Watch and Reserve antibiotics.

**Conclusion:**

Our multicentre study across three sites in Indonesia revealed neonatal sepsis is predominantly caused by Gram-negative pathogens, with very poor coverage provided by currently recommended empiric regimens. A high burden of multidrug-resistant pathogens drives empirical use of broad-spectrum antibiotics. There is an urgent need for new antibiotic regimens and infection prevention and control strategies to treat neonatal sepsis in regions with a high prevalence of multidrug-resistant infections.

**WHAT IS ALREADY KNOWN ON THIS TOPIC**

- There is mounting evidence to suggest neonatal sepsis is increasingly caused by multidrug-resistant bacteria in resource-constrained healthcare settings, particularly in Southeast Asia.
- There are sparse published granular data evaluating the epidemiology of neonatal sepsis, and susceptibility (or otherwise) to empiric antibiotic regimens in many of these high-burden countries.

**WHAT THIS STUDY ADDS**

- We conducted a systematic evaluation of 2 years of blood culture data, to summarise the pathogens (and antimicrobial susceptibility profiles) causative of neonatal sepsis across three major urban tertiary hospitals in Indonesia. Concurrently, we evaluated antibiotic prescribing patterns via a point prevalence survey.
- A Bayesian-weighted incidence syndromic combination antibiogram was built to provide coverage estimates for frequently prescribed antimicrobial regimens used to treat neonatal sepsis.

**HOW THIS STUDY MIGHT AFFECT RESEARCH, PRACTICE OR POLICY**

- Our multicentre study revealed alarming rates of non-susceptibility to currently recommended antimicrobial regimens for hospitalised neonates with sepsis in Indonesia.
- Results from this study highlight an urgent need for prioritised development of antibiotics to treat neonatal sepsis in high-burden settings.

## Introduction

 Despite progress in reducing child mortality over recent decades, neonatal mortality rates have improved minimally, with 3.9 million cases of neonatal sepsis occurring globally each year.^1^ In the context of rising antimicrobial resistance (AMR), this burden is further increasing.^2^ Neonatal sepsis is currently responsible for over half a million deaths each year, and survivors are also at risk of long-term morbidity—including a higher rate of postdischarge mortality and long-term cognitive and physical impairment.^3^

Marked disparities in the burden of neonatal sepsis are evident worldwide, with 85% of sepsis-related deaths occurring in low and middle-income countries, particularly in sub-Saharan Africa and Southeast Asia.^4^ Rising rates of neonatal multidrug-resistant (MDR) neonatal infections are particularly evident in the populous nations of Southeast Asia, in the context of rapidly increasing facility births alongside advancing medical capacity, which results in prolonged hospitalisation of prematurely born babies.^5 6^ High rates of neonatal sepsis due to gram-negative bacteria in these settings suggests a significant burden of horizontal transmission may be driving the rising rates of MDR infections.^7^

Indonesia is the world’s fourth most populous nation, with over 4.2 million births occurring each year and an infant mortality rate of 19 per 1000 live births, which is higher than the Southeast Asia and Pacific average.^8^ Despite this, there remains limited published data on the burden of neonatal sepsis in Indonesia, of particular concern given the suggestions of high rates of AMR within the region.^9^ Small observational studies from Indonesian referral hospitals have revealed neonatal sepsis incidence rates ranging from 9% to 30%, with a mortality rate of 12%–50%.^9 10^

In resource-constrained healthcare settings, antibiotic non-susceptibility rates have dramatically increased in the past two decades, further complicating neonatal sepsis management.^6 11^ Across South Asian countries, there are high rates of non-susceptibility to the currently recommended WHO empirical regimen of ampicillin and gentamicin,^5^ and neonatal sepsis due to MDR pathogens is driving worsening case fatality rates.^12 13^

The treatment of neonatal sepsis requires the selection of empirical regimens likely to be efficacious, as microbiological investigations can be challenging in this age group, providing limited opportunity for pathogen-directed treatment.^11 14^ In the context of rising AMR, ensuring empirical regimens to treat neonatal sepsis is efficacious and is of significant importance given the morbidity and mortality burden that may result from inadequately treated neonatal sepsis. While local antibiograms can help provide ‘bug-drug’ pathogen susceptibility rates, a weighted incidence syndromic combination antibiogram (WISCA)—which utilises Bayesian methods to calculate the weighted coverage estimate for prespecified antibiotic regimens for specific clinical syndromes–can provide more useful coverage estimates to guide the likelihood that a specific clinical syndrome is expected to be treated with specified antibiotic regimens when the causative pathogen is not known.^15^

The aim of this multicentre study was to establish high-quality observational data on the epidemiology and burden of AMR in neonatal sepsis in Indonesia and to use these data to develop a WISCA to guide clinicians and policymakers with coverage estimates of commonly recommended empirical antibiotic regimens to treat neonatal sepsis. A secondary aim was to evaluate local antibiotic prescribing patterns via a point prevalence prescribing survey. These results can subsequently guide interventional and efficacy-based trials to improve neonatal sepsis outcomes in high-burden neonatal sepsis settings.

## Methods

### Study design

To enable comparable data to that published by other informative neonatal sepsis observational studies,^16^ we followed similar methodology and conducted a multicentre, retrospective observational study of the causes of neonatal bloodstream infections at three tertiary referral hospitals across major urban centres in Indonesia: the National Referral Hospital, Dr Cipto Mangunkusumo (Jakarta); Hasan Sadikin Hospital (Bandung) and Dr Soetomo Hospital (Surabaya). The sites were specifically selected for their large number of births, paucity of published data on the causative pathogens responsible for neonatal sepsis and concerns regarding rising rates of MDR pathogens limiting efficacious treatment options.^10 17^ Concurrently, an antibiotic point prevalence survey (PPS) was undertaken to assess antibiotic prescribing patterns in each hospital.

### Data collection

Microbiological data were systematically reviewed to identify all neonates ≤28 days of age with positive blood cultures recorded during a predefined 2-year period: 1 January 2019 to 31 December 2020. Data on the total number of positive blood cultures collected over this period, the number of positive neonatal blood cultures and the number of blood cultures with significant (bacterial or fungal) pathogens isolated within the 2-year period were systematically extracted. Any blood culture from the same patient that was positive for the same pathogen within 4 weeks of the originally reported blood culture was considered a duplicate and removed from the analysis.

To understand local practices that might be contributing to the local MDR infection prevalence, antibiotic prescribing practices were evaluated via a PPS. All data were anonymised and uploaded onto a secure REDCap database.

### Ethical approval

Ethical approval was obtained from Universitas Indonesia, Jakarta Institutional Research Board (21-06-0674); Dr Soetomo Hospital, Surabaya (006/Komitlitkes/2022) and Komite Etik Penelitian RSUP Dr Hasan Sadikin, Bandung (23-05-0858), with a waiver of consent (including parental consent) provided.

At all sites, the indication for neonatal blood culture collection was clinical instability or transfer from a peripheral facility due to clinical deterioration. Blood cultures are routinely performed at microbiology laboratories with a BacT/ALERT 3D-automated microbial detection system (bioMerieux, Durham, USA). Isolated pathogens were identified using conventional identification methods and VITEK-2 (bioMerieux, Durham, USA), and susceptibility testing was undertaken using disc diffusion methods according to Clinical and Laboratory Standards Institute (CLSI) standards.^18^

### Statistical analysis

Prescribing patterns from the antibiotic prescribing PPS were evaluated to ascertain the proportion of ‘Access, Watch and Reserve’ antibiotics prescribed across the three clinical sites.^19^ For the neonatal sepsis epidemiological data, descriptive tables were produced summarising causative pathogen prevalence and AMR across the three sites combined. Susceptibility to three antimicrobial regimens was evaluated: (a) aminopenicillin plus gentamicin (recommended by WHO as first-line treatment for neonatal sepsis), (b) non-anti-Pseudomonal third-generation cephalosporins: ceftriaxone/cefotaxime (recommended by WHO as second-line treatment for neonatal sepsis) and (c) carbapenems (a ‘Watch’ antibiotic that is frequently empirically prescribed in Southeast Asia due to increasing resistance to currently recommended regimens).^16 19^ Isolates were classified as either susceptible or resistant, with intermediate susceptibilities considered resistant. Not all causative pathogens underwent susceptibility testing for each of these regimens, thus the proportion resistant was calculated from the number tested for each antimicrobial agent (table 1; online supplemental table 1).

**Table 1:** Non-susceptibility of tested bacterial isolates causative of neonatal sepsis across three sites in Indonesia

	Aminopenicillins:*proportion resistant(%, n/N)	Gentamicin:proportion resistant (%, n/N)	Third-generation cephalosporins:†proportion resistant(%, n/N)	Carbapenems:proportion resistant (%, n/N)
Gram-negative pathogens				
* Acinetobacter* spp.	IR	90% (131/145)	IR	79% (121/153)
* Citrobacter* spp.	0 (0/2)	0 (0/2)	0 (0/2)	0 (0/2)
* E. coli*	68% (34/50)	72% (36/50)	31% (16/52)	32% (16/50)
* Enterobacter* spp.	78% (47/60)	55% (36/66)	62% (34/55)	13% (9/67)
* Klebsiella* spp.	IR	87% (237/271)	90% (241/269)	20% (57/281)
* Non-typhoidalSalmonella*spp*.*	100% (13/13)	100% (13/13)	100% (13/13)	0 (0/13)
*Proteus* spp.	50% (1/2)	0 (0/2)	50% (1/2)	0 (0/2)
*Pseudomonas* spp.	IR	29% (7/24)	IR	31% (8/26)
* Salmonella* typhi	0 (0/1)	100% (1/1)	0 (0/1)	0 (0/1)
* Serratia* spp	50% (7/14)	43% (6/14)	43% (6/14)	0 (0/15)
Gram-positive pathogens				
* Enterococcus* spp.	14% (2/14)	IR	IR	78% (7/9)
Group B Streptococcus	NT	NT	10% (1/10)	NT
* S. aureus*	14% (1/7)	NT	NT	NT

IR: presumed intrinsic resistance, as per EUCAST guidelines.^20^

EUCAST: European Committee on Antimicrobial Susceptibility Testing

* Aminopenicillins = amoxicillin and/or ampicillin.

† Non-anti-pseudomonal third-generation cephalosporins (ceftriaxone/cefotaxime).

IR, intrinsically resistant; NT, not tested.

WISCA antibiograms were fit using a Bayesian framework to estimate expected coverage of each of the three antimicrobial regimens across all causative pathogens. The expected coverage of each regimen is a function of the estimated prevalence of causative pathogens and the estimated susceptibility of each pathogen to that regimen. In this Bayesian framework, the observed pathogen data were assumed to be drawn from a multinomial distribution with a non-informative Dirichlet(1,1,1,…,1) prior. For each pathogen, the proportion susceptible to each regimen was assumed to be drawn from a binomial distribution with a beta prior. An uninformative Beta(1,1) prior was used for most; however, for pathogens expected to have intrinsic resistance to the regimen, a beta(1,9999) prior was used, to dominate any sensitivity results, following previously published assumptions for developing a WISCA model.^15^ For the combined aminopenicillin plus gentamicin regimen, the antimicrobial with higher susceptibility for any particular pathogen was used to reflect the overall regimen susceptibility for that pathogen. As per European Committee on Antimicrobial Susceptibility Testing (EUCAST) criteria,^20^ intrinsic resistance to third-generation cephalosporins was assumed for *Acinetobacter* spp, *Pseudomonas* spp and *Enterococcus* spp; and for *Staphylococcus aureus*, third-generation cephalosporin and meropenem susceptibility was derived from information on methicillin resistance. Susceptibility to aminopenicillin plus gentamicin and carbapenem regimens was assumed for *Streptococcus agalactiae* (n=12). Fungal species, coagulase-negative Staphylococci (CoNS) and pathogens without susceptibility information were excluded from WISCA models. Models were run for 100 000 iterations with 10 000 burn-in. Convergence was checked through visual inspection of trace plots.^15^

### Patient and public involvement

The research question was co-designed with clinicians and researchers working in high-burden neonatal sepsis settings across Indonesia. The findings of this research are informing prospective surveillance studies to reduce the burden of hospital-acquired infections in neonatal sepsis. As these data are retrospectively collated on discharged patients, patients were not burdened by the intervention or in time required to participate in the research. The findings of this study will be shared at international conferences to promote enhanced research focused on reducing the burden of morbidity and mortality due to neonatal sepsis in high-burden AMR settings.

## Results

### Antibiotic prescribing patterns

Over the study period, the three urban tertiary hospitals included in this study had high numbers of neonatal admissions each year: 1433 in Jakarta, 399 in Bandung, and 1490 in Surabaya. The empiric antibiotic regimen for neonates with early-onset sepsis (EOS, defined as sepsis within the first 72 hours of life) is ampicillin and gentamicin across all sites. For late-onset sepsis (LOS), Surabaya follows the same regimen, while in Jakarta local guidelines recommend ampicillin-sulbactam plus gentamicin and in Bandung cefotaxime is recommended. Empirical guidelines for the treatment of neonatal meningitis were ampicillin-sulbactam plus gentamicin in Jakarta, meropenem in Bandung, and cefotaxime plus gentamicin in Surabaya. Full neonatal intensive care support was available at all sites, including mechanical ventilation and inotrope support.

**Figure 1:**
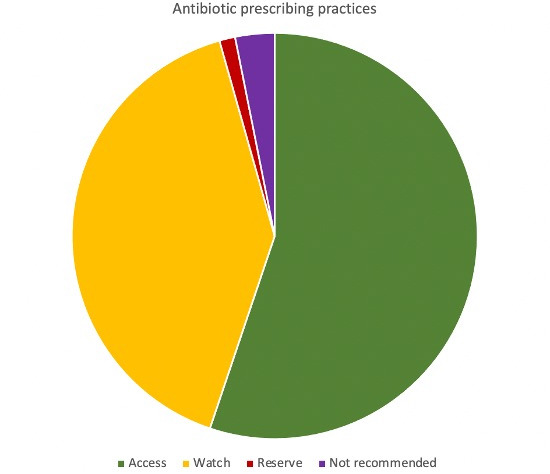
Antibiotic prescribing based on the WHO AWaRe (‘Access’, ‘Watch’ and ‘Reserve’)^21^ categories across all three sites.

The median gestational age of admissions on the day of the antibiotic PPS (undertaken in the second half of 2022) was 31 weeks in Jakarta (IQR 30–33), 36 weeks in Bandung (IQR 34–38) and 31 weeks in Surabaya (IQR 34.5–38). The median number of antibiotics prescribed was two for each neonate admitted across all sites on the day of the PPS; with 55%, 40% and 1% of all antibiotics prescribed classified as WHO ‘Access’, ‘Watch’ and ‘Reserve’ antibiotics. A further ten antibiotic prescriptions were from WHO ‘not recommended’ categories (all pertaining to cefoperazone-sulbactam) (figure 1).^21^

### Blood culture analyses and coverage provided by commonly-prescribed antibiotic regimens

**Figure 2:**
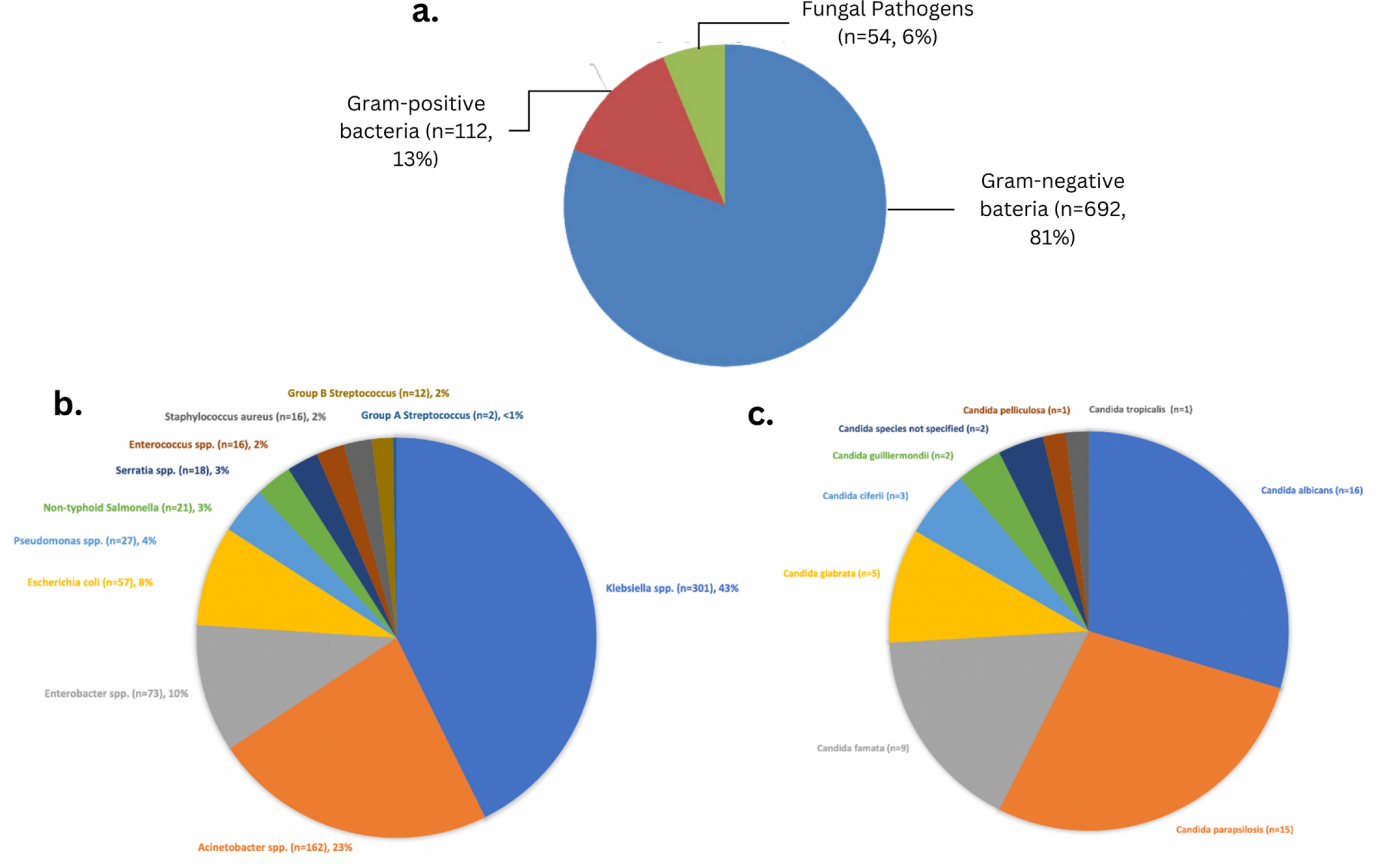
Pathogens responsible for neonatal sepsis across three Indonesian tertiary referral hospitals; (**a**) overall pathogen breakdown; (**b**) by gram-positive and gram-negative bacterial genera; (**c**) invasive fungal infections. NB: coagulase-negative *Staphylococci* (CoNS), *Corynebacterium* (Diphtheroids) spp., *Micrococcus* spp. *Propionibacterium* spp. and *Bacillus* spp., were removed as presumed contaminants. There were also a small number of cases due to *Proteus* spp. (n=3), *Salmonella* serovar Typhi (n=2), *Citrobacter* spp. (n=2) and other gram-negative bacilli (unspecified, n=26) not included in figure 2b.

A total of 5439 blood culture isolates from neonates with clinically-suspected sepsis were analysed from the three study sites: 2059 from Jakarta, 1731 from Surabaya and 1649 from Bandung. Significant pathogens were cultured from 16% (n=858) of all isolates. As clinical information was limited, CoNS were not included in the list of significant pathogens. Overall, 81% (n=692) of pathogens isolated were Gram-negative bacteria, 13% (n=112) were Gram-positive bacteria and 6% (n=54) were *Candida* spp (figure 2).

*Klebsiella* spp was the most commonly identified pathogen responsible for neonatal sepsis across all sites (35%, n=301/858). The next most prevalent pathogens were all Gram-negative bacteria: *Acinetobacter* spp (19%, n=162/858), *Enterobacter* spp (9%, n=73/858), *Escherichia coli* (7%, n=57/858) and *Pseudomonas* spp (3%, n=27/858) (figure 2). Unlike its predominance in high-income healthcare settings, Group B *Streptococcus* (GBS) was only responsible for 12 cases of neonatal sepsis, while *S. aureus* was responsible for 16 cases (2%; figure 2). Among fungal pathogens, critically important WHO Priority Pathogen^22^ *Candida albicans* accounted for the majority of cases of candidaemia (n=16/54, 30%) (figure 2). Fluconazole susceptibility among all *Candida* spp isolates was high (30 susceptible out of 33 tested, 90%).

**Figure 3:**
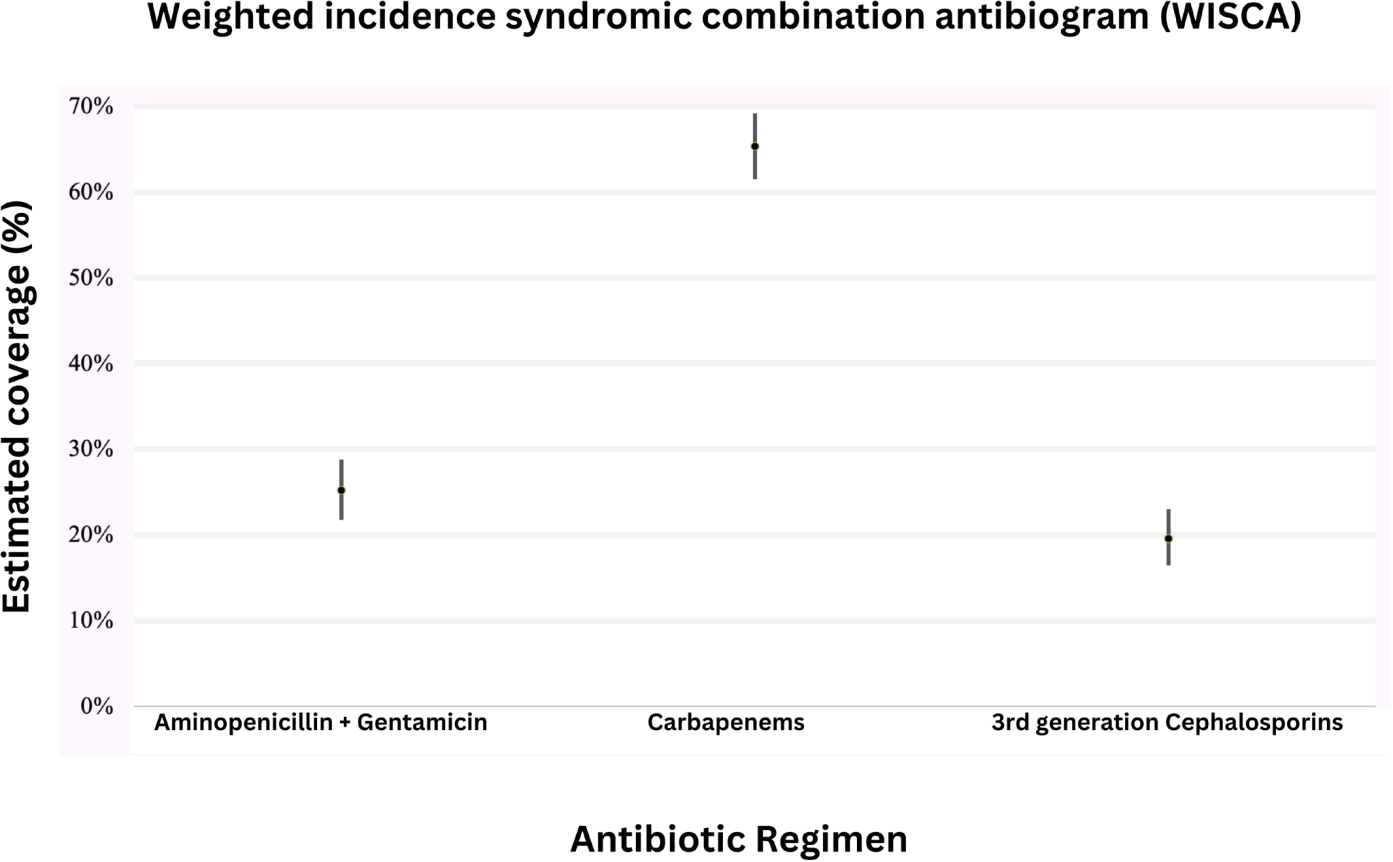
Mean estimated coverage provided by commonly-prescribed antimicrobial regimens to treat neonatal sepsis. The solid points indicate median estimates, with surrounding bars indicating 95% credible intervals.

A total of 710 isolates with susceptibility data were included in the Bayesian WISCA model (table 1; online supplemental table 1). Model convergence was achieved with acceptable autocorrelation margins. Overall, 54 *Candida* spp, 259 CoNS and 94 pathogen isolates without susceptibility data were excluded from the model. Figure 3 shows the estimated coverage of each of the three regimens with 95% credible intervals. Estimated coverage was 25% (95% credible interval (CI) 22 to 29%) to aminopenicillin plus gentamicin, 20% (95% CI 17% to 23%) to third-generation cephalosporins, and 65% (95% CI 62% to 69%) to carbapenems (figure 3).

When analysed by pathogen, estimated coverage for *Klebsiella* spp was most adequate with carbapenems (80%, 95% CI 75% to 84%), with much lower coverage provided by aminopenicillin plus gentamicin (13%, 95% CI 9% to 17%) and third-generation cephalosporins (11%, 95% CI 7% to 15%). For *Acinetobacter* spp*,* only 21% coverage was provided by carbapenems (95% CI 15% to 28%), and even less by aminopenicillins plus gentamicin (10%, 95% CI 6% to 15%).

## Conclusion

Our multicentre study has revealed alarming rates of non-susceptibility to commonly prescribed antimicrobials to treat neonatal sepsis in hospital settings in Indonesia, largely driven by a high prevalence of Gram-negative bacteria as causative pathogens. These data align with recent epidemiological studies conducted in other settings across Asia,^23^,^25^ and globally,^11^ highlighting the urgent need for new antibiotic regimens to treat neonatal sepsis in settings with a high burden of MDR infections.^6^

The evolving resistance profile of pathogens within these urban tertiary hospital centres reveals a growing departure from susceptibility to the currently recommended WHO empirical treatment regimens for neonatal sepsis as well as to ‘Watch’ antimicrobial agents such as meropenem. Our WISCA model estimates that WHO-recommended first-line and second-line antibiotic regimens to treat neonatal sepsis provide coverage below 30% in hospitalised neonates in Indonesia. These high rates of MDR infections force clinicians to prescribe broad-spectrum agents, including those in the WHO ‘Watch‘ and ‘Reserve’ list (alongside ‘not recommended’ antibiotics), in an effort to improve treatment efficacy in the context of high rates of local non-susceptibility. While our findings estimate higher coverage for carbapenems, which are already used as an empirical regimen in one site within our study, it is important to note that overuse of carbapenems is not just expensive to healthcare settings but also will further propagate Gram-negative resistance mechanisms.^26 27^

Our data are limited by their ascertainment from urban teaching hospitals, likely overestimating the AMR patterns of bacteria causing neonatal sepsis in Indonesia, as these tertiary referral hospitals would have a high burden of hospital-acquired infections. Our study is also limited by its retrospective nature and microbiological focus, which limits an understanding of clinical outcomes associated with resistance profiles reported; yet the morbidity and mortality impact of culture-positive neonatal sepsis, particularly when caused by MDR pathogens, has already been proven to be significant.^11^

The high rates of AMR revealed by our study have prompted the commencement of systematic prospective (clinical and microbiological) surveillance studies at these sites, including studies to evaluate colonisation dynamics of MDR pathogens in neonatal intensive care units. Furthermore, the data revealed by this study are also important in informing interventional trials for new antibiotic regimens to treat neonatal sepsis in settings with a high prevalence of MDR pathogens.^28^ However, in the context of the significant (and rising) burden of AMR evident in neonatal sepsis globally, it is concerning that so few clinical trials evaluating improved treatment options for neonatal sepsis are currently recruiting.^2 29^ Research is urgently needed to build on the limited data pertaining to the pharmacokinetics, safety and efficacy of both newer and older agents that might be efficacious in treating neonatal sepsis in the context of rising AMR globally.

The high rates of carbapenem resistance among *Klebsiella* spp and *Acinetobacter* spp in this study support the urgent need to prioritise the development of antibiotics that provide efficacy against these pathogens, responsible for an increasingly high proportion of neonatal sepsis cases.^30 31^ The highest prevalence of *Acinetobacter* spp as a causative pathogen for neonatal sepsis was found at the tertiary referral hospital in Jakarta, where ampicillin-sulbactam and gentamicin are utilised as empiric therapy for late-onset neonatal sepsis. This empiric prescribing guideline reflects the challenge for local clinicians in choosing efficacious agents that might reduce the overwhelming burden of neonatal mortality due to MDR infections in their facility, but concurrently might drive further selection of these bacteria within the neonatal intensive care environment.^7^

The causative pathogens responsible for neonatal sepsis in our study contrast starkly with the pathogen distribution of neonatal sepsis in high-income countries, where GBS continues to be the most common cause of neonatal sepsis—a pathogen far easier to treat due to its high susceptibility to currently recommended antibiotics for neonatal sepsis and meningitis.^32^ The epidemiological shift from a predominance of Gram-negative pathogens causing neonatal sepsis to Gram-positive pathogens (such as GBS or CoNS) requires both healthcare and community development, including access to clean water and sanitation in the community and stringent infection, prevention and control practices within the hospital environment.^33^ In the populous nations in Southeast Asia, where the number of facility births is increasing rapidly yet the availability of nurses and midwives declines due to the ‘brain drain’ to the Global North, a persistent burden of Gram-negative sepsis is likely to continue to prevail—and is now further complicated by the alarming rise of AMR.^27^

Furthermore, the high prevalence of critically important fungal priority pathogens in our study reveals the significant burden of Candidaemia in neonatal sepsis in Indonesia. While fluconazole susceptibility was high within isolated *Candida* spp overall, the prevalence of this pathogen highlights the need to consider invasive fungal infections as a causative pathogen in unstable neonates with clinical sepsis in high-burden settings.^34^

This multicentre study conducted in Indonesia reveals an alarming burden of Gram-negative bacteria as causative pathogens in neonatal sepsis, resulting in vastly inefficacious coverage provided by WHO-recommended antibiotics to treat neonatal sepsis. The marked discrepancy between the susceptibility profiles of the most common causes of neonatal sepsis in Indonesia and the currently recommended antibiotic agents to treat neonatal sepsis reflects the local need to prescribe ‘Watch’, ‘Reserve’ and ‘not commenced’ antibiotics, as observed in our study. This research adds to the growing evidence that neonatal sepsis in resource-constrained settings is commonly caused by MDR Gram-negative pathogens and there is a crucial need to enhance the availability of infection, prevention and control strategies while concurrently enhancing research focused on identifying new antibiotic regimens with improved efficacy to treat neonatal sepsis. As resource-constrained healthcare settings enhance their capacity to provide life-saving care to prematurely born neonates, resulting in prolonged hospital stays of critically ill neonates, the nosocomial acquisition of MDR Gram-negative bacteria needs to be closely monitored. Streamlined development of prioritised antibiotics for this vulnerable population needs urgent global attention to reduce the currently unnecessary morbidity and mortality caused by neonatal sepsis globally.^2 27 31^

## Supplementary material

10.1136/bmjgh-2024-016272 online supplemental file 1

10.1136/bmjgh-2024-016272 online supplemental file 2

## Data Availability

Data are available upon reasonable request.
