# Supplementary material for: Epidemiology of sepsis in hospitalised neonates in Indonesia: high burden of multidrug-resistant infections reveals poor coverage provided by recommended antibiotic regimens
Source: BMJ Glob Health. 2025 Apr 9;10(4):e016272. doi: 10.1136/bmjgh-2024-016272 (PMC11987146; doi:10.1136/bmjgh-2024-016272)
Supplement: online supplemental file 1 [file bmjgh-10-4-s001.docx]

**Author Reflexivity Statement:**

1. **How does this study address local research and policy priorities?**

Clinicians working in Indonesia have noted increasingly difficult to treat neonatal infections, and would like to evaluate the epidemiology and susceptibility of their burden of disease against global guidelines. These data will also ensure antibiotic development can be focused on addressing the current epidemiology of AMR in neonatal sepsis in high-burden regions.

1. **How were local researchers involved in study design?**

The first author is a paeditric infectious diseases clinician in Jakarta; whilst other authors are clinicians and microbiologists working in Indonesia. Supported by protocols designed in collaboration with other global neonatal sepsis studies, they felt the proposed methodology would enable AMR in neonatal sepsis to be evaluated in a pragmatic and cost-effective way.

1. **How has funding been used to support the local research team?**

This project has enabled further grants which have enabled the commencement of a project to understand the transmission of hospital-acquired infections in Indonesia supporting a large local team in Indonesia.

1. **How are research staff who conducted data collection acknowledged?**

All those involved in data collection and manuscript feedback are included as authors.

1. **Do all members of the research partnership have access to study data?**

All members of the partnership have access to data.

1. **How was data used to develop analytical skills within the partnership?**

Local researchers worked with Univesity of Sydney statisticians and mathematicians to build the WISCA model.

1. **How have research partners collaborated in interpreting study data?**

Local researchers worked with Univesity of Sydney statisticians and mathematicians to build the WISCA model. They felt the findings reflected what they see in their day-to-day clinical practice.

1. **How were research partners supported to develop writing skills?**

The research team writing this statement is predominantly composed of clinicians and academics with already strong research skills, who were further supported by the senior authors who have published extensively in this field (Sharland and Williams).

1. **How will research products be shared to address local needs?**

This study has enabled the co-design of subsequent studies to reduce the burden of AMR in neonatal sepsis in Indonesia, currently recruiting.

1. **How is the leadership, contribution and ownership of this work by LMIC researchers recognised within the authorship?**

The local study team are recognized as senior and named authors.

1. **How have early career researchers across the partnership been included within the authorship team?**

We have included early career researchers (ND, BD, MH) across both HIC and LMIC within the authorship team.

1. **How has gender balance been addressed within the authorship?**

We have a good balance of male and female authors across the cited authorship list, from both HIC and LMICs.

1. **How has the project contributed to training of LMIC researchers?**

Research funding leveraged as part of this project will continue to support employment of the research team in Indonesia who were part of this study, and future co-designed studies.

1. **How has the project contributed to improvements in local infrastructure?**

This project has enabled the design of prospective surveillance studies and enhanced microbiological surveillance of neonatal infections.

1. **What safeguarding procedures were used to protect local study participants and researchers?**

Data were anonymized and collated prior to analyses.
