# Supplementary material for: Epidemiology of sepsis in hospitalised neonates in Indonesia: high burden of multidrug-resistant infections reveals poor coverage provided by recommended antibiotic regimens
Source: BMJ Glob Health. 2025 Apr 9;10(4):e016272. doi: 10.1136/bmjgh-2024-016272 (PMC11987146; doi:10.1136/bmjgh-2024-016272)
Supplement: online supplemental file 2 [file bmjgh-10-4-s002.pdf]

Supplementary Table 1. Proportion of non-susceptible isolates (by species, antimicrobial and clinical site) included in the WISCA model

	Aminopenicillin^ Proportion resistant (%) (n/N)				Gentamicin Proportion resistant (%) (n/N)				3rd Generation Cephalosporin* Proportion resistant (%) (n/N)				Carbapenem Proportion resistant (%) (n/N)			
	**Jakarta**	**Bandung**	**Surabaya**	**Total**	**Jakarta**	**Bandung**	**Surabaya**	**Total**	**Jakarta**	**Bandung**	**Surabaya**	**Total**	**Jakarta**	**Bandung**	**Surabaya**	**Total**
Gram-negative																
Acinetobacter spp.	IR	IR	IR	IR	85.71 (66/77)	96.49 (55/57)	90.91 (10/11)	90.34 (131/145)	IR	IR	IR	IR	80 (68/85)	82.46 (47/57)	54.55 (6/11)	79.08 (121/153)
Citrobacter spp.	0 (0/2)	NT	NT	0 (0/2)	0 (0/2)	NT	NT	0 (0/2)	0 (0/2)	NT	NT	0 (0/2)	0 (0/2)	NT	NT	0 (0/2)
E. coli	47.06 (8/17)	100 (26/26)	0 (0/7)	68 (34/50)	52.94 (9/17)	88.46 (23/26)	57.14 (4/7)	72 (36/50)	47.37 (9/19)	11.54 (3/26)	57.14 (4/7)	30.77 (16/52)	29.41 (5/17)	34.62 (9/26)	28.57 (2/7)	32 (16/50)
Enterobacter spp.	76.36 (42/55)	100 (5/5)	NT	78.33 (47/60)	60 (33/55)	0 (0/5)	50 (3/6)	54.55 (36/66)	59.09 (26/44)	100 (5/5)	50 (3/6)	61.82 (34/55)	14.29 (8/56)	0 (0/5)	16.67 (1/6)	13.43 (9/67)
Klebsiella spp.	IR	IR	IR	IR	87.03 (161/185)	94.34 (50/53)	78.79 (26/33)	87.45 (237/271)	86.34 (158/183)	100 (53/53)	90.91 (30/33)	89.59 (241/269)	24.1 (47/195)	7.55 (4/53)	18.18 (6/33)	20.28 (57/281)
Non-typhoid Salmonella	NT	100 (13/13)	NT	100 (13/13)	NT	100 (13/13)	NT	100 (13/13)	NT	100 (13/13)	NT	100 (13/13)	NT	0 (0/13)	NT	0 (0/13)
Proteus spp.	100 (1/1)	0 (0/1)	NT	50 (1/2)	0 (0/1)	0 (0/1)	NT	0 (0/2)	100 (1/1)	0 (0/1)	NT	50 (1/2)	0 (0/1)	0 (0/1)	NT	0 (0/2)
Pseudomonas spp.	IR	IR	IR	IR	20 (3/15)	40 (2/5)	50 (2/4)	29.17 (7/24)	IR	IR	IR	IR	29.41 (5/17)	40 (2/5)	25 (1/4)	30.77 (8/26)
Salmonella typhi	NT	0 (0/1)	NT	0 (0/1)	NT	100 (1/1)	NT	100 (1/1)	NT	0 (0/1)	NT	0 (0/1)	NT	0 (0/1)	NT	0 (0/1)
Serratia spp.	50 (1/2)	100 (6/6)	0 (0/6)	50 (7/14)	100 (2/2)	50 (3/6)	16.67 (1/6)	42.86 (6/14)	100 (2/2)	50 (3/6)	16.67 (1/6)	42.86 (6/14)	0 (0/3)	0 (0/6)	0 (0/6)	0 (0/15)
Gram-positive																
Enterococcus spp.	25 (2/8)	0 (0/6)	NT	14.29 (2/14)	IR	IR	IR	IR	IR	IR	IR	IR	77.78 (7/9)	NT	NT	77.78 (7/9)
Group B Streptococcus	NT	NT	NT		NT	NT	NT	NT	100 (1/1)	0 (0/9)	NT	10 (1/10)	NT	NT	NT	NT
S. aureus	20 (1/5)	NT	0 (0/2)	14.29 (1/7)	NT	NT	NT	NT	NT	NT	NT	NT	NT	NT	NT	NT

*Non-anti-Pseudomonal third-generation cephalosporins (ceftriaxone/cefotaxime); ^Aminopenicillins = amoxicillin and/or ampicillin; IR: presumed intrinsic resistance, as per EUCAST guidelines;[20] NT: not tested; IR = intrinsically resistant.
